# Non-Isothermal Free-Surface Viscous Flow of Polymer Melts in Pipe Extrusion Using an Open-Source Interface Tracking Finite Volume Method

**DOI:** 10.3390/polym13244454

**Published:** 2021-12-19

**Authors:** Célio Fernandes, Ahmad Fakhari, Željko Tukovic

**Affiliations:** 1 Institute for Polymers and Composites (IPC), Department of Polymer Engineering, Engineering School of the University of Minho, Campus of Azurém, 4800-058 Guimarães, Portugal; 2 Transport Phenomena Research Center (CEFT), Mechanical Engineering Department, Faculty of Engineering of the University of Porto, Rua Dr. Roberto Frias s/n, 4200-465 Porto, Portugal; ahmadfakhari@gmail.com; 3 Faculty of Mechanical Engineering and Naval Architecture, University of Zagreb, Ulica Ivana Lučića 5, 10000 Zagreb, Croatia; Zeljko.Tukovic@fsb.hr

**Keywords:** extrudate swell, pipe die, polymer melt, Herschel–Bulkley fluids, yield stress, finite volume method, interface tracking, free-surfaces, OpenFOAM

## Abstract

Polymer extrudate swelling is a rheological phenomenon that occurs after polymer melt flow emerges at the die exit of extrusion equipment due to molecular stress relaxations and flow redistributions. Specifically, with the growing demand for large scale and high productivity, polymer pipes have recently been produced by extrusion. This study reports the development of a new incompressible non-isothermal finite volume method, based on the Arbitrary Lagrangian–Eulerian (ALE) formulation, to compute the viscous flow of polymer melts obeying the Herschel–Bulkley constitutive equation. The Papanastasiou-regularized version of the constitutive equation is employed. The influence of the temperature on the rheological behavior of the material is controlled by the Williams–Landel–Ferry (WLF) function. The new method is validated by comparing the extrudate swell ratio obtained for Bingham and Herschel–Bulkley flows (shear-thinning and shear-thickening) with reference data found in the scientific literature. Additionally, the essential flow characteristics including yield-stress, inertia and non-isothermal effects were investigated.

## 1. Introduction

The extrudate swell flow is a well-known benchmark problem in the polymer processing field, where a free-surface and a boundary stress singularity at the die exit are present. The variety of solutions for free-surface flows is usually very limited to simple cases [1]. However, with the increasing demand for large diameters and high productivity, plastic pipes have recently been produced [2].

The polymer production process is mainly non-isothermal in nature, including heat transfer phenomena such as heating and cooling, e.g., in the injection molding [3,4] or extrusion [5,6,7,8] processes. Since the flow properties are strongly dependent on rheology and temperature, it is of great interest to understand and predict this type of heat transfer phenomenon. Although there is increasing research effort on non-isothermal free-surface flows, these studies are limited to academic purposes and problems. Extrudates in general can swell up to 15% due to thermal effects [9,10]. Phuoc and Tanner [11] developed a finite element scheme based on the Galerkin method to explore the effects of thermally induced property changes in extrusion. The swelling of self-heating extruded jets was investigated, and a new phenomenon, the so-called thermal extrudate swell, was discovered. Extruded expansions of up to 70% of the die diameter have been found in a Newtonian fluid with thermal properties similar to those of low density polyethylene. Subsequently, Karagiannis et al. [12] developed a three-dimensional (3D) non-isothermal code to study viscous free-surface flows with exponential dependence of viscosity on temperature. Again, the code was based on Galerkin’s finite-element formulation, and, apart from the phenomenon of thermally induced extrudate swelling, the bending and distortion of the extrudate due to temperature differences and/or geometric asymmetries were confirmed numerically and experimentally. Finally, Spanjaards et al. [13] developed a 3D transient non-isothermal finite element code to predict the extruded shape of viscoelastic fluids emerging from an asymmetric keyhole-shaped die. A systematic study was carried out to decouple the effects of shear-thinning, elasticity, and temperature in the shape of the extrudate.

Another important effect to be considered when predicting the swelling of the extrudate is the inertia, through the dimensionless Reynolds number, Re, defined as the ratio of inertial forces to viscous forces within a fluid that is subjected to relative internal movement due to different fluid velocities. In Newtonian flow, the extrudate swell ratio, χ, defined as the ratio of the final extrudate radius to that of the die, decreases with increasing Re and finally achieves a contraction rather than swelling, since χ is less than unity [14,15]. An important class of materials commonly used in extrusion is the yield-stress fluid, which does not deform when subjected to a stress below the yield stress, $\tau_{0}$. Bingham [16] developed the simplest constitutive equation with a yield-stress, which was then combined with a power-law model to account for the effects of shear-thinning or shear-thickening, known as the Herschel–Bulkley model [17]. To solve fluid flows described by constitutive equations with unyielded $\left(\tau \leq \tau_{0}\right)$ and yielded regions $\left(\tau >\tau_{0}\right)$, a special approach to overcome the numerical difficulties created by these models (the viscosity diverges to infinity as the strain rate $\dot{\gamma }$ approaches zero) must be adopted. For that purpose, a common methodology is to do a regularization of the constitutive equation so that the same expression is uniformly valid for any value of $\dot{\gamma }$, i.e., to ensure that the viscosity is a continuous function of strain rate. Notice that here τ and $\dot{\gamma }$ represent the magnitudes of the stress tensor τ and rate-of-strain tensor $\dot{\gamma }$, respectively. Papanastasiou [18] developed the regularization model for the Bingham constitutive equation, which was then extended to work with the Herschel–Bulkley model by Ellwood et al. [19]. A survey of regularization models used in the scientific literature can be found in Frigaard and Nouar [20]. For creeping flow conditions (Re≪1), there are several studies where the results for the extrudate swell of materials with yield stress are presented [18,21,22,23,24]. Generally, the conclusion is that in creeping extrudate swell flow of yield-stress materials, the extrudate swell ratio decreases as yield stress increases. Additionally, it was noticed that beyond a certain value of $\tau_{0}$, the swell contracts slightly (χ<1) and reaches a minimum, from which it starts to increase again to reach unity asymptotically. For moderate Reynolds numbers (1<Re<200), the scientific literature devoted to the study of the effects of inertia on the extrudate swell of yield-stress fluids is scarce [19,25]. In this situation, as the yield stress increases, swelling at lower Reynolds numbers and contraction at higher Reynolds numbers are reduced.

In recent decades, Computational Fluid Dynamics (CFD) simulation has expanded as a significant framework to help engineers understand and improve polymer processing techniques. Nonetheless, the current state of the art of numerical methods for complex problems, which is the case of non-isothermal free-surface extrudate swell flow of yield-stress fluids, is insufficient for practical needs. In addition, proprietary packages are expensive and cannot be modified to account for the engineer’s needs. As the solver here presented is based in an open-source finite volume computational library, OpenFOAM [26], this allows more capabilities to be added to the code to take into account the physics involved in the phenomenon of interest, thereby making the simulation more realistic. Therefore, a general and freely available solver, based on the open-source framework OpenFOAM [26], for the simulation of the non-isothermal free-surface flow of yield-stress fluids would be of great importance. Hence, this work deals with the development of a solver to calculate non-isothermal free-surface flows of yield-stress fluids as an extension of the isothermal free-surface flow solver presented elsewhere [15,27]. Notice that in Fakhari et al. [15], we developed the predecessor of the current code, which was used to describe Newtonian isothermal fluid flows, and there the efficiency of this framework was demonstrated in terms of both considering a larger computational time-step and also the smaller CPU wall time to perform each iteration of the simulation. The objective of this work is to investigate the combined effects of inertia, yield-stress, and temperature on the extrudate swell of a pipe flow. It is worth pointing out that although the melted polymer chain strongly depends on shear and stretch [28], this work focuses on the macroscale dimensional distribution of the melted polymer in the extruded swell phenomena and does not go through the microscale changes of the material such as crystallization kinetics.

The paper is organized as follows: in Section 2, we present the description of the problem, specifically, the balance equations that govern the non-isothermal free-surface flow of generalized Newtonian fluids, the boundary and initial conditions, and the Arbitrary Lagrangian–Eulerian formulation for the mesh movement. In Section 3, the numerical method used to implement the proposed model is described. In Section 4, we present the numerical results obtained for the non-isothermal extrudate swell of the Herschel–Bulkley yield-stress fluid model, taking into account the combined effects of inertia, yield-stress and temperature. Finally, in Section 5, we summarize the main conclusions of this work.

## 2. Problem Description

In this section, the governing equations to solve the incompressible and non-isothermal polymer-melt extrudate flow in dies of cylindrical shape for pipe production are described.

### 2.1. Balance Equations

The equations of motion governing the non-isothermal flow of incompressible generalized Newtonian fluids inside an arbitrary volume *V* bounded by a closed moving surface *S* include continuity, momentum, and energy equations, which can be written as:

(1) $$\begin{array}{c} \nabla \cdot u=0, \end{array}$$

(2) $$\begin{array}{c} \frac{\partial u}{\partial t}+\nabla \cdot \left((u-u_{S})u\right)=-\nabla P+\nabla \cdot \tau , \end{array}$$

(3) $$\begin{array}{c} \rho \;c_{p}\;\left(\frac{\partial T}{\partial t}+u\cdot \nabla T\right)=k_{p}\nabla^{2}T+\tau :D, \end{array}$$

where ∇ is the Hamilton differential operator, u is the fluid velocity vector, $u_{S}$ is the velocity of surface *S*, *P* is the kinematic pressure, τ is the deviatoric stress tensor, ρ is the fluid density, $c_{p}$ is the heat capacity, *T* is the temperature, $k_{p}$ is the isotropic thermal conductivity based on the heat flux Fourier’s law, and $D=(\nabla u+\nabla u^{T})/2$ is the strain rate tensor. Note that the last term on the right hand side of Equation (3) represents viscous heat dissipation and is written in a form that assumes all mechanical energy is dissipated as heat.

### 2.2. Constitutive Equation

The following constitutive equation is adopted to describe the relationship between stress and strain for the viscous fluid flow,

(4) $$\begin{array}{c} \tau =2\eta (\dot{\gamma })D, \end{array}$$

where $\dot{\gamma }=\sqrt{2D:D}$ is the shear rate (invariant of the strain rate tensor **D**) and $\eta (\dot{\gamma })$ is the apparent viscosity, which is described by the Herschel–Bulkley model [17] with the Papanastasiou regularization [18,19],

(5) $$\begin{array}{c} \eta \left(\dot{\gamma }\right)=\min\left\{\eta_{0},\tau_{0}{\dot{\gamma }}^{-1}\left[1-\exp\left(-m\dot{\gamma }\right)\right]+k{\dot{\gamma }}^{n-1}\right\}, \end{array}$$

where *k* is the consistency constant of proportionality, *n* is the flow index exponent, which measures the degree to which the fluid is shear-thinning or shear-thickening, *m* is a stress growth parameter, and $\tau_{0}$ is the yield shear stress. Note that the original Papanastasiou regularization does not include the artificial upper-bounding by $\eta_{0}$. However, this bounding is needed to avoid an infinite viscosity for $\dot{\gamma }\to 0$ and n<1. The Herschel–Bulkley model is reduced to the Newtonian model when the yield-stress is zero, i.e., $\tau_{0}=0$, and the flow index exponent is one, i.e., n=1.

### 2.3. Temperature Dependency of the Apparent Viscosity

To analyze the effect of the temperature dependency of the apparent viscosity on hydrodynamic and thermal behaviour of the flow, we keep constant the flow index *n* and the yield stress $\tau_{0}$ and consider only a temperature-dependent consistency k(T):

(6) $$\begin{array}{c} k\left(T\right)=a_{T}\left(T\right)k_{0}, \end{array}$$

where $a_{T}$ denotes the shift factor and $k_{0}$ is the consistency viscosity at the reference temperature $T_{0}$. The shift factor $a_{T}$ is defined by the Williams–Landel–Ferry (WLF) function:

(7) $$\begin{array}{c} \log\left(a_{T}\right)=\frac{-c_{1}\left(T-T_{0}\right)}{c_{2}+T-T_{0}}, \end{array}$$

where $c_{1}$ and $c_{2}$ are material parameters.

### 2.4. Boundary and Initial Conditions

Considering that the fluid phases are immiscible, the fluid flow Equations (1)–(3) can be used for each phase individually, and at the interface, the proper boundary conditions must be used. First, the kinematiccondition states that the fluid velocities on the two sides of the interface, $u_{1f}$ and $u_{2f}$, must be continuous

(8) $$\begin{array}{c} u_{1f}=u_{2f}. \end{array}$$

Then, from the momentum conservation law, Equation (2), follows the dynamiccondition, which states that forces acting on the fluid at the interface are in equilibrium,

(9) $$\begin{array}{c} \left[T_{2}-T_{1}\right]\cdot n=\nabla_{s}\sigma -\sigma \kappa n, \end{array}$$

where $T_{1}$ and $T_{2}$ are the stress tensors defined in terms of the local fluid pressure and velocity fields as $T_{1}=-p_{1}I+\eta_{1}\left(\dot{\gamma }\right)\left[\nabla u_{1}+{\left(\nabla u_{1}\right)}^{T}\right]$ and $T_{2}=-p_{2}I+\eta_{2}\left(\dot{\gamma }\right)\left[\nabla u_{2}+{\left(\nabla u_{2}\right)}^{T}\right]$, respectively, σ is the interfacial tension and $\nabla_{s}=\left[I-\mathrm{nn}\right]\cdot \nabla =\nabla -n\frac{\partial }{\partial n}$ is the tangential gradient operator, which appears because σ and n are defined only on the surface. From Equation (9) we derive the normal and tangential force balances [29] appropriate at a fluid–fluid interface,

(10) $$\begin{array}{c} p_{2}-p_{1}=\sigma \kappa -2\left(\eta_{2}\left(\dot{\gamma }\right)-\eta_{1}\left(\dot{\gamma }\right)\right)\nabla_{s}\cdot u, \end{array}$$

(11) $$\begin{array}{cc} \;\eta_{2}\left(\dot{\gamma }\right)\left[n\cdot {\left(\nabla u_{t}\right)}_{2}\right]-\eta_{1}\left(\dot{\gamma }\right)\left[n\cdot {\left(\nabla u_{t}\right)}_{1}\right]= & -\nabla_{s}\sigma \\ & -n\left(\eta_{2}\left(\dot{\gamma }\right)-\eta_{1}\left(\dot{\gamma }\right)\right)\nabla_{s}\cdot u\\ & -\left(\eta_{2}\left(\dot{\gamma }\right)-\eta_{1}\left(\dot{\gamma }\right)\right)\left(\nabla_{s}u\right)\cdot n, \end{array}$$

where $\kappa =-\nabla_{s}\cdot n$ is twice the mean curvature of the interface and $u_{t}=\left(I-nn\right)\cdot u$ is the tangential velocity component.

Dirichlet or Neumann boundary conditions for temperature *T* are specified depending on the boundary wall considered. Natural convection on boundaries are treated by setting the gradient (Neumann type boundary condition) according to

(12) $$\begin{array}{c} \nabla T_{b}=\frac{h}{k_{p}}\left(T_{b}-T_{\infty }\right), \end{array}$$

where the temperature on the boundary $T_{b}$ is obtained from the previous iteration, *h* is the convection heat transfer coefficient, and $T_{\infty }$ is the ambient temperature.

### 2.5. Arbitrary Lagrange–Eulerian Formulation

The above mathematical model, valid for arbitrary moving volume, is obtained from the corresponding material volume model using the Reynolds’ transport theorem. For an arbitrary moving volume, the relationship between the rate of change of the volume *V* and the velocity $u_{s}$ is defined by the geometrical(space)conservationlaw [30],

(13) $$\begin{array}{c} \frac{d}{dt}\int_{V}dV-\oint_{S}n\cdot u_{s}\;dS=0. \end{array}$$

The problem of extrudate swelling contains moving boundaries due to the movement of the free surfaces of the extrudate. Thus, the domain is described with a mesh that is moving in time in such a way that the mesh moves with the free surfaces.

## 3. Numerical Method

The mathematical model for the non-isothermal free-surface flow of an incompressible generalized Newtonian fluid described in Section 2 is numerically discretized using the finite volume method [27]. First, the numerical integration in time is performed using a second-order accurate implicit method [31], referred to as the backward scheme. Next, the integral forms of the fluid flow equations are discretized in space using a second-order accurate cell-centred unstructured finite volume method. The spatial domain is discretized using a mesh, which is constituted by finite volumes (the so-called cells or elements) with an arbitrary volume *V* bounded by a closed moving surface *S*, that conserve the relevant quantities, such as mass, momentum, and energy:

(14) $$\begin{array}{c} \oint_{S}n\cdot u\;dS=0, \end{array}$$

(15) $$\begin{array}{c} \frac{d}{dt}\int_{V}u\;dV+\oint_{S}n\cdot \left(u-u_{S}\right)u\;dS=-\int_{V}\nabla P\;dV+\oint_{S}n\cdot \tau \;dS, \end{array}$$

(16) $$\begin{array}{c} \rho c_{p}\left(\frac{d}{dt}\int_{V}T\;dV+\oint_{S}n\cdot \left(uT\right)\;dS\right)=\oint_{S}n\cdot \left(k_{p}\nabla T\right)\;dS+\int_{V}\left(\tau :D\right)\;dV. \end{array}$$

Detailed information of the finite volume discretization employed in the moving mesh interface tracking algorithm can be found in Tuković and Jasak [27]. To summarize, the surface integrals of an integral conservation equation are transformed into sums of face integrals which together with the volume integrals are approximated to second-order accuracy by using the mid-point rule. Therefore, Equations (14)–(16) for each cell are written as,

(17) $$\begin{array}{c} \sum_{f}n_{f}^{n}\cdot u_{f}^{n}\;S_{f}^{n}=0, \end{array}$$

(18) $$\begin{array}{c} \frac{3u_{P}^{n}V_{P}^{n}-4u_{P}^{o}V_{P}^{o}+u_{P}^{oo}V_{P}^{oo}}{2\Delta t}+\sum_{f}\left({\dot{m}}_{f}^{n}-{\dot{U}}_{f}^{n}\right)u_{f}^{n}=-{\left(\nabla P\right)}_{P}^{n}V_{P}^{n}+\sum_{f}n_{f}^{n}\cdot \tau_{f}^{n}S_{f}^{n}, \end{array}$$

(19) $$\begin{array}{cc} \rho_{P}{\left(c_{p}\right)}_{P}\frac{3T_{P}^{n}V_{P}^{n}-4T_{P}^{o}V_{P}^{o}+T_{P}^{oo}V_{P}^{oo}}{2\Delta t}+\sum_{f}\rho_{f}{\left(c_{p}\right)}_{f}n_{f}^{n}\cdot u_{f}^{n}T_{f}^{n}S_{f}^{n}= & \sum_{f}{\left(k_{p}\right)}_{f}n_{f}^{n}\cdot {\left(\nabla T\right)}_{f}^{n}S_{f}^{n}\\ & +\left(\tau_{P}^{n}:D_{P}^{n}\right)V_{P}^{n}, \end{array}$$

where Δt is the time-step, the subscripts *P* and *f* represent the cell-center and face-center values at cell with volume $V_{P}$, the superscripts *n*, *o* and oo represent values evaluated at the new time instance $t^{n}$ and two previous time instances $t^{o}$ and $t^{oo}=t^{o}-\Delta t$. Finally, the cell-face mass flux ${\dot{m}}_{f}^{n}=n_{f}^{n}\cdot u_{f}^{n}S_{f}^{n}$ must satisfy the discretized mass conservation law (Equation (17)), and the face volume flux ${\dot{U}}_{f}^{n}$ must satisfy the discretized geometrical conservation law (Equation (13)). Equation (17) is linearized for a pressure correction according to the consistent non-iterative PISO algorithm [32,33,34]. The algorithm can be summarized as follows [35]:

1. Set initial guess of the solution at time *t* for pressure, velocity, temperature and mass flow rate fields $p^{n}$, $u^{n}$, $T^{n}$, and ${\dot{m}}_{f}^{n}$, respectively.
2. Define displacement directions for the interfacial mesh points and the control points.
3. In order to compensate the net mass flux through the interface, calculate displacement of the interface mesh points (the least-squares volume-point interpolation scheme was employed [36]).
4. Displacement of the interface mesh points is used as a boundary condition for the solution of the mesh motion problem. After mesh movement, the new face volume fluxes ${\dot{U}}_{f}^{n}$ are calculated.
5. Update pressure and velocity boundary conditions at the interface.
6. Assemble and solve *implicitly* the momentum equation given by Equation (18) to obtain a new velocity field $u^{*}$.
7. Compute the mass flow rate at the cell faces ${\dot{m}}_{f}$ using the Rhie–Chow interpolation technique [37].
8. Using the new mass flow rates computed in Step 7, assemble the pressure correction equation (Equation (17)) and solve it to obtain a pressure correction field $p^{\prime }$.
9. Update the pressure and velocity fields at the cell centroids, $p^{n}$ and $u^{*}$, respectively, and correct the mass flow rate at the cell faces ${\dot{m}}_{f}^{*}$, to obtain continuity-satisfying fields $p^{*}$, $u^{**}$ and ${\dot{m}}_{f}^{**}$. The consistent version of the SIMPLE (Semi Implicit Method for Pressure Linked Equations) algorithm is used here by assuming that the velocity correction at point *P* is the weighted average of the corrections at the neighboring grid points [32,33,34], resulting in a better estimating for the velocity corrections, and consequently, a higher rate of convergence is obtained [15].
10. Using the latest available velocity and pressure fields, $u^{**}$ and $p^{*}$, respectively, assemble and solve *explicitly* the momentum equation to obtain a new velocity field $u^{***}$.
11. Update the mass flow rate at the cell faces ${\dot{m}}_{f}^{***}$ using the Rhie–Chow interpolation technique [37].
12. Using the new mass flow rates computed in Step 11, assemble the pressure correction equation and solve it to obtain a pressure correction field $p^{\prime \prime }$.
13. Update the pressure and velocity fields at the cell centroids, $p^{*}$ and $u^{***}$, respectively, and correct the mass flow rate at the cell faces ${\dot{m}}_{f}^{***}$, to obtain $p^{**}$, $u^{****}$ and ${\dot{m}}_{f}^{****}$.
14. Go to Step 10 and repeat for a given number of corrector steps (nCorrectors=3 in this work).
15. Solve the temperature equation, Equation (19), to obtain $T^{*}$ and update the temperature-dependent consistency viscosity Equations (6) and (7).
16. Set the initial guess for pressure, velocity, temperature and mass flow rate as $p^{n}=p^{**}$, $u^{n}=u^{****}$, $T^{n}=T^{*}$ and ${\dot{m}}_{f}^{n}={\dot{m}}_{f}^{****}$, respectively.
17. Repeat from Step 3 for a given number of times (nOuterCorrectors=10 in this work).
18. Set the converged solution at time t=t+Δt and advance to the next time step.
19. Return to Step 1 and repeat until the last time step is reached.

The Poisson-type equation for pressure is solved with a conjugate gradient method with Cholesky preconditioner and the velocity and temperature linear systems are solved using BiCGstab with an Incomplete Lower-Upper (ILU) preconditioning [38,39,40].

## 4. Results and Discussion

In this section, we present the validation and assessment of the newly developed moving mesh finite-volume interface tracking solver that is able to efficiently handle inelastic non-Newtonian matrix-based free-surface flows with non-isothermal effects. In this work, the fluid rheology is described by the Herschel–Bulkley constitutive equation with the Papanastasiou regularization. The newly developed solver is tested against fluid flow simulations in an axisymmetric domain geometry typical of pipe extrusion. The first case study is devoted to the extrudate swell of a Bingham fluid, and subsequently, the extrudate swell of Herschel–Bulkley shear-thinning and shear-thickening flows is presented. These studies aim to verify the solver’s capabilities to accurately predict the extrudate swell of inelastic non-Newtonian matrix-based fluids. Additionally, the effects of inertia and yield stress on the extrudate swell are investigated. Finally, the non-isothermal extrudate swell effects are studied for a Bingham fluid at moderate Reynolds number (Re=10), in order to test the robustness of the newly developed numerical algorithm, specifically for non-isothermal calculations.

### 4.1. Problem Domain and Meshes

**Figure 1:**
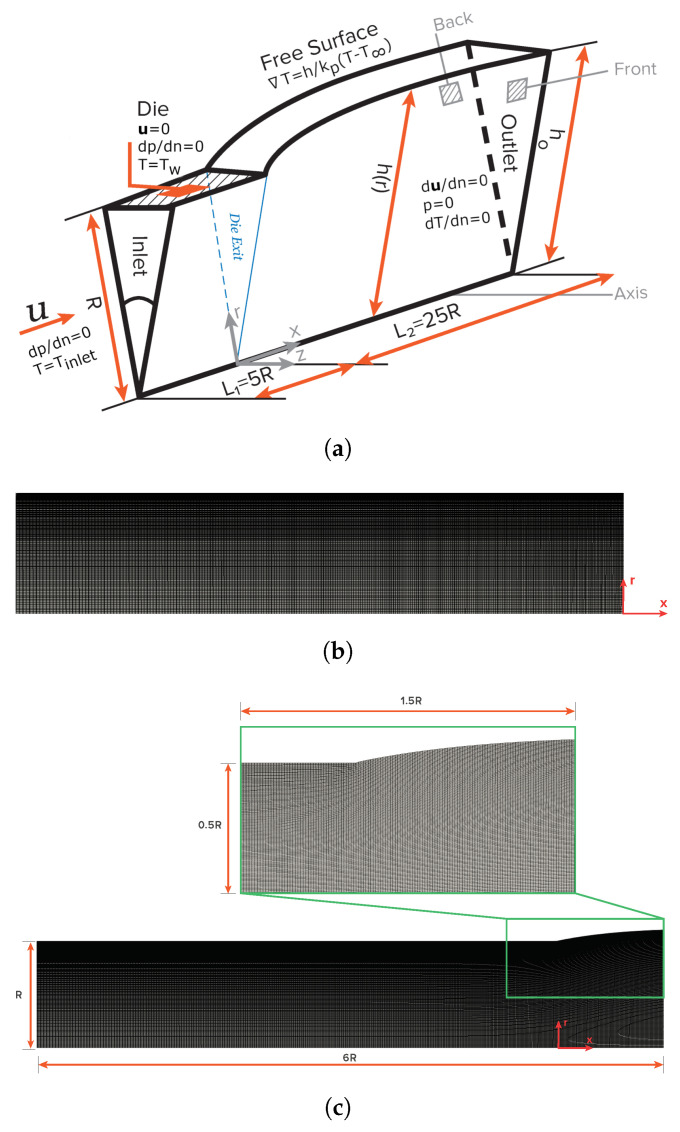
Schematic representation of (**a**) the axisymmetric extrudate swell domain geometry and boundary faces (**b**) of an indicative discretization mesh at the initial time step t=0, and (**c**) at steady state.

The benchmark case study that will be discussed is the axisymmetric extrudate swell of non-Newtonian inelastic fluids. A schematic representation of the computational flow domain, the boundary faces, and the discretization mesh for the initial time step (t=0) and at a steady state is shown in Figure 1. The level of mesh refinement used in the numerical studies carried out in this study corresponds to the same level employed in mesh M5 of our previous work [15], which resulted from a mesh convergence analysis. Polar coordinates are employed for the description of the axisymmetric flow domain, thus x=(r,z). The half width of the axisymmetric channel is denoted as *R*, which is considered to be the scaling length. The inlet plane is taken sufficiently far upstream from the exit so that the flow is fully developed with a mean velocity *U*. In the axisymmetric domain, the two lateral boundary sides are considered to be wedge patches (i.e., the cylinder is specified as a wedge of small angle, e.g., 5°, and a thickness cell running along the plane of symmetry, encompassing one of the coordinate planes). At the bottom, the axis of symmetry is considered as empty patch. At the solid die wall, the no-slip (tangential velocity is zero) and no-penetration (normal velocity is zero) conditions are imposed for velocity, zero-gradient pressure, and a fixed value temperature. At the free-surface, the kinematic condition, Equation (8), and the dynamic condition, Equations (10) and (11), are imposed for pressure and velocity, along with natural convection, Equation (12), for temperature. Finally, the outflow plane is taken sufficiently far downstream of the die exit so that the flow is uniform. The die exit of the axisymmetric domain is located at x=5R from the inlet, and the outflow is located at x=25R from the die exit.

The dimensionless numbers governing the flow are the Reynolds number,

(20) $$\begin{array}{c} Re=\frac{\rho U^{2-n}R^{n}}{k}, \end{array}$$

which is the ratio of inertial forces to viscous forces within a fluid that is subject to relative internal movement due to different fluid velocities; the Bingham number,

(21) $$\begin{array}{c} Bn=\frac{\tau_{0}R^{n}}{kU^{n}}, \end{array}$$

which is the ratio of yield stress to viscous stress and describes the extent to which the controllable yield stress can exceed the viscous stress; the dimensionless growth exponent,

(22) $$\begin{array}{c} M=\frac{mU}{R}, \end{array}$$

that, by using a regularized constitutive equation such as the Papanastasiou model, it will determine up to which convergent results can be obtained (in our simulations M≥500 for 0<Bn≤10); and the Prandtl number,

(23) $$\begin{array}{c} Pr=\frac{kc_{p}}{k_{p}}{\left(\frac{U}{R}\right)}^{n-1}, \end{array}$$

which is the ratio of moment diffusivity (kinematic viscosity) and thermal diffusivity of a fluid, expressing the relationship between the movement quantity diffusion and the heat quantity diffusion within the fluid itself.

### 4.2. The Effects of Inertia and Yield-Stress in the Extrudate Swell of Bingham Fluids

**Figure 2:**
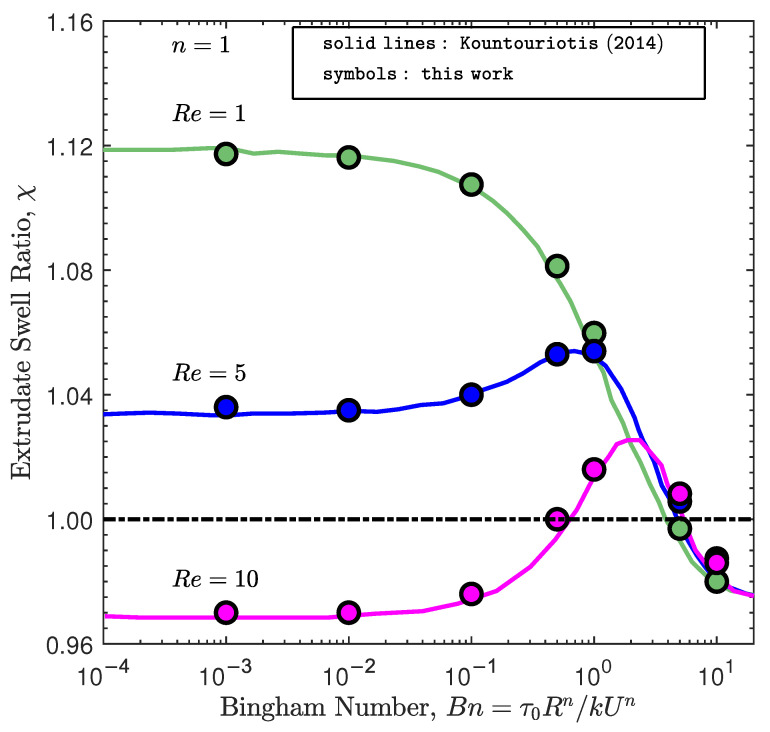
Steady-state extrudate swell ratio χ for the isothermal extrudate swell of Bingham fluids (n=1) at Re={1,5,10} and $Bn=\{10^{-3},10^{-2},10^{-1},0.5,1,5,10\}$. Solid lines represent the results obtained by Kountouriotis et al. [41], and the symbols represent the results obtained by the newly developed interface tracking code.

Initially, the combination of inertia and yield-stress effects on the extrudate swell ratio obtained for the isothermal flow of Bingham fluids (i.e., n=1) is studied. The swell ratio is defined as the height of the free-surface away from the die exit, where the plug flow has been established, divided by the die radius, i.e., $\chi =h_{0}/R$. As shown in Figure 2, the extrudate swell ratio obtained by the newly developed solver is very close to the results presented by Kountouriotis et al. [41] at Re=1,5, and 10. For the lowest Reynolds number (Re=1), as the yield stress effect is enhanced (i.e., increase of the Bn number), the extrudate swell shrinks steeply for Bn>0.1, and above a critical value of Bn>5, the swell contracts and becomes smaller than unity. At Re=5 the extrudate swell ratio increases with increasing Bn, reaching a maximum at Bn≈1. Subsequently, the extrudate swell ratio decreases and becomes lower than unity for Bn>5. At Re=10, the extrudate swell ratio is below one for the lowest Bn numbers (Bn<0.5), and then it enlarges, being higher than one as Bn enhances, reaching a maximum at Bn≈2. After that the swell ratio χ diminishes and again becomes lower than unity for Bn>5. Regardless of the Re number used in the simulations, for Bn≥5, the extrudate swell ratio is very similar on each curve, which means that the yield stress effects are predominant compared to the inertial effects.

**Figure 3:**
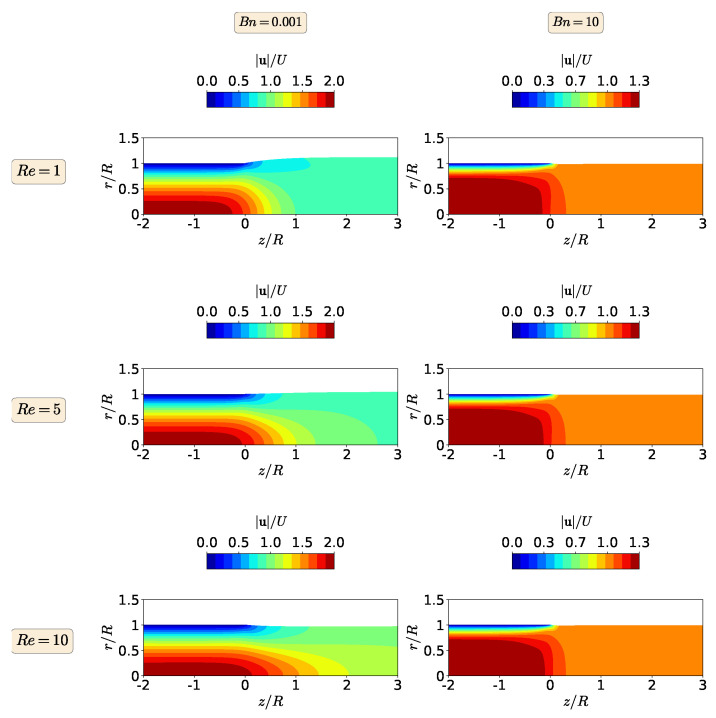
Contours of the magnitude of the polymer velocity vector at steady-state for the isothermal extrudate swell of Bingham fluids (n=1) when Re=1 (**top**), Re=5 (**middle**) and Re=10 (**bottom**), and Bn=0.001 (**left**) and Bn=10 (**right**).

In Figure 3, the magnitude of the polymer velocity vector is shown for Re=1, 5, and 10 and Bn=0.001 and 10. At Bn=0.001 (negligible yield-stress effects), the maximum value of the magnitude of the polymer velocity vector is twice the inlet velocity and the enhancement of inertia effects through the increase in the Re number leads to the reduction of the die swell ratio, mimicking the behavior of the extrudate swell for a Newtonian fluid, as presented by Fakhari et al. [15]. When the highest Bn number is considered (Bn=10), the maximum value of the magnitude of the polymer velocity vector is 1.3 times the inlet velocity. This means that the increase in the yield stress promotes the retardation of the flow, changing from parabolic shape profile to plug-flow. The consequence is that the extrudate swell ratio decreases and, in fact, contracts. Note that the contour plots of the magnitude of the polymer velocity vector are very similar at this higher Bingham number, where increasing inertia effects do not change the shape of the flow field.

**Figure 4:**
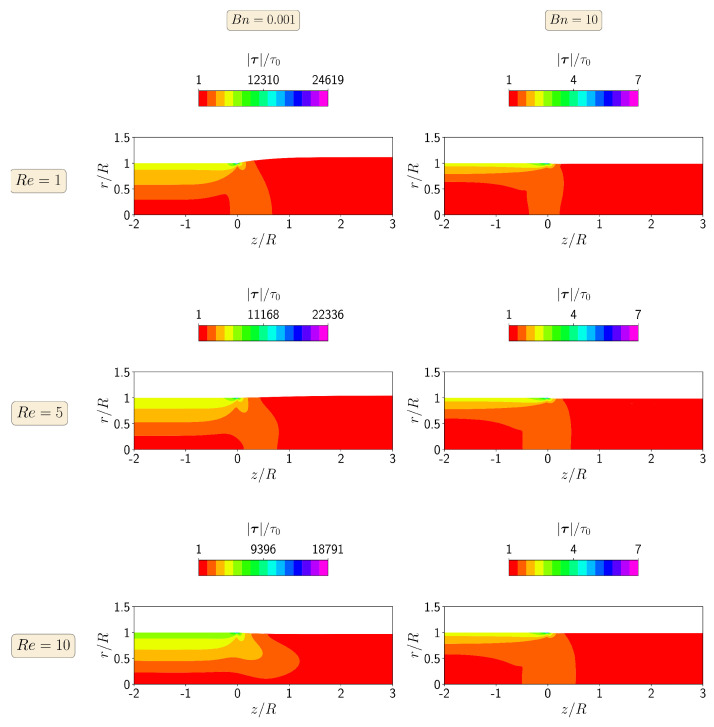
Contours of the magnitude of the polymer stress tensor at steady-state for the isothermal extrudate swell of Bingham fluids (n=1) when Re=1 (**top**), Re=5 (**middle**) and Re=10 (**bottom**), and Bn=0.001 (**left**) and Bn=10 (**right**).

Figure 4 shows the contours of the magnitude of the polymer stress tensor at steady-state for Re=1, 5, and 10 at Bn=0.001 and 10. When the yield stress effects are negligible (Bn=0.001), the maximum value of the stress tensor magnitude occurs for the lowest Reynolds number (Re=1), corresponding to the highest extrudate swell ratio. Increasing the inertia effects decreases the localized maximum value of the magnitude of the polymer stress tensor in the top right corner of the die wall, leading to a decrease in the extrudate swell ratio. On the other hand, when the yield stress effects dominate the flow (i.e., Bn=10), the maximum value of the magnitude of the polymer stress tensor is constant for all Re numbers from 1 to 10, being three orders of magnitude lower than the case with Bn=0.001.

### 4.3. The Effects of Inertia and Yield-Stress in the Extrudate Swell of Herschel–Bulkley Fluids

**Figure 5:**
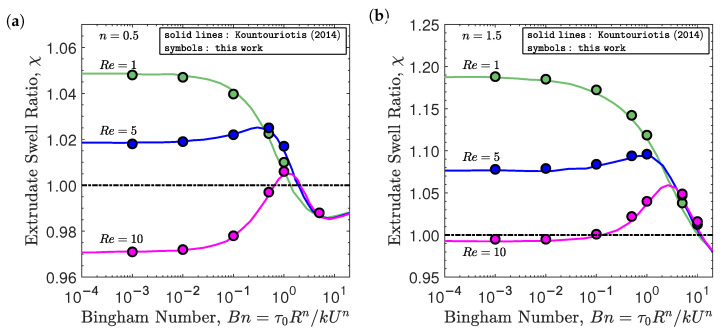
Steady-state extrudate swell ratio χ for the isothermal extrudate swell of Herschel–Bulkley fluids at Re={1,5,10} and $Bn=\{10^{-3},10^{-2},10^{-1},0.5,1,5,10\}$ with (**a**) n=0.5 and (**b**) n=1.5. Solid lines represent the results obtained by Kountouriotis et al. [41], and the symbols represent the results obtained by the newly developed interface tracking code.

In this section, the effects of inertia and yield-stress are investigated on the isothermal extrudate swell of Herschel–Bulkley flows (n≠1). For this purpose, the simulations were carried out at the same Re and Bn numbers as those presented in Section 4.2, but with a flow index exponent representative of shear-thinning, n=0.5, and shear-thickening, n=1.5, behaviors. As shown in Figure 5, the extrudate swell ratio obtained by the newly developed solver is again very close to the results presented by Kountouriotis et al. [41] for both n=0.5 and n=1.5 at Re=1,5, and 10. The general trend of the shear-thinning extrudate swell ratio (see Figure 5a) at the different Re numbers is similar to the behavior shown for the Bingham flow (n=1) in Figure 2. In Figure 5a, we can see that, in general, for n=0.5, the extrudate swell ratio is lower than the ones obtained for n=1 and n=1.5. This behavior allows us to conclude that the shear-thinning nature of the Herschel–Bulkley fluid helps to reduce the swelling of the polymer. On the other hand, for the shear-thickening behavior (n=1.5), the extrudate swell ratio at different Re and Bn numbers is the highest, as shown in Figure 5b. Additionally, the variation in the extrudate swell ratio with the increase in the yield stress effects is more abrupt for this shear-thickening fluid, reaching a maximum of 20% at Re=1.

**Figure 6:**
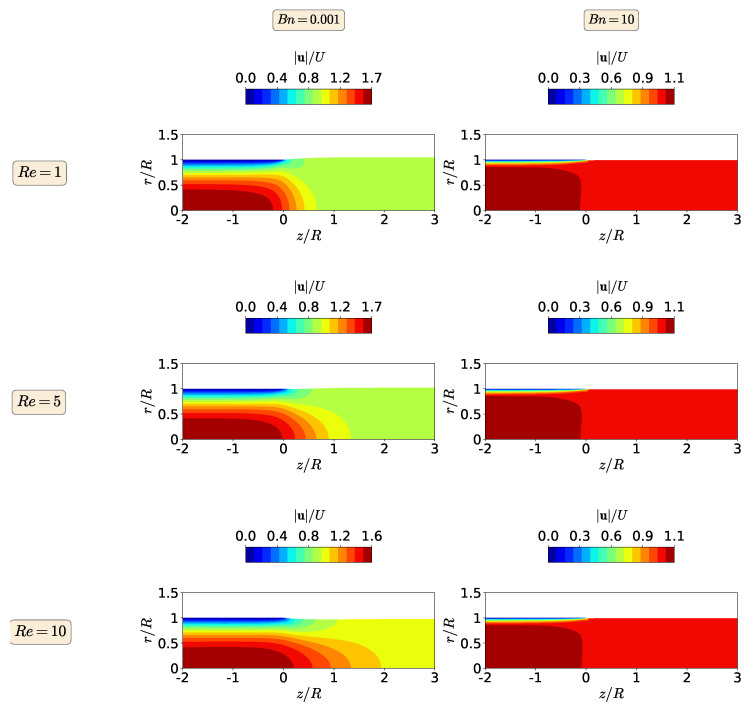
Contours of the magnitude of the polymer velocity vector at steady-state for the isothermal extrudate swell of shear-thinning Herschel–Bulkley fluids (n=0.5) when Re=1 (**top**), Re=5 (**middle**) and Re=10 (**bottom**), and Bn=0.001 (**left**) and Bn=10 (**right**).

Figure 6 shows the contours of the magnitude of the polymer velocity vector for the isothermal extrudate swell of shear-thinning Herschel-Bulkley fluids (n=0.5), when Re=1,5 and 10 and Bn=0.001s and 10. At Bn=0.001 (negligible yield stress effects), the maximum value of the magnitude of the polymer velocity vector is approximately 1.7 times the inlet velocity for 1≤Re≤10, which is 15% smaller than the value obtained for the Bingham flow (n=1), leading to variations of 7% in the extrudate swell ratio when increasing Re from 1 to 10. On the other hand, when the highest Bn number is considered (Bn=10), the maximum value for the magnitude of the polymer velocity vector is 1.1 times the inlet velocity, which is again 15% smaller than the value obtained for the Bingham flow (n=1). However, for this strongly dominated yield stress flow, the extrudate swell ratio is kept constant for 1≤Re≤10 and again with a plug-flow velocity profile leading to swell contraction (χ<1).

**Figure 7:**
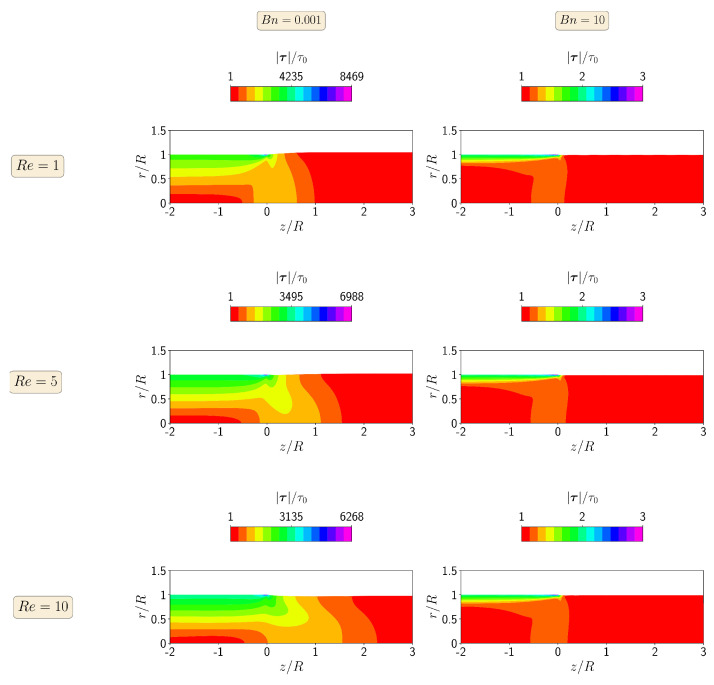
Contours of the magnitude of the polymer stress tensor at steady state for the isothermal extrudate swell of shear-thinning Herschel–Bulkley fluids (n=0.5) when Re=1 (**top**), Re=5 (**middle**) and Re=10 (**bottom**), and Bn=0.001 (**left**) and Bn=10 (**right**).

In Figure 7, we show the contour plots of the magnitude of the polymer stress tensor at steady-state for the isothermal extrudate swell of shear-thinning Herschel–Bulkley fluids (n=0.5) when Re=1,5, and 10 and Bn=0.001 and 10. When the yield stress effects are negligible (Bn=0.001), the maximum value of the magnitude of the polymer stress tensor decreases with the increase of inertia from Re=1 to 10, while at the highest Bn number, the inertia do not have influence on the maximum value of the magnitude of the polymer stress tensor. In general, for both of the Bn numbers, the magnitudes of the polymer stress tensor are approximately one third of the ones obtained for the Bingham fluid (n=1).

**Figure 8:**
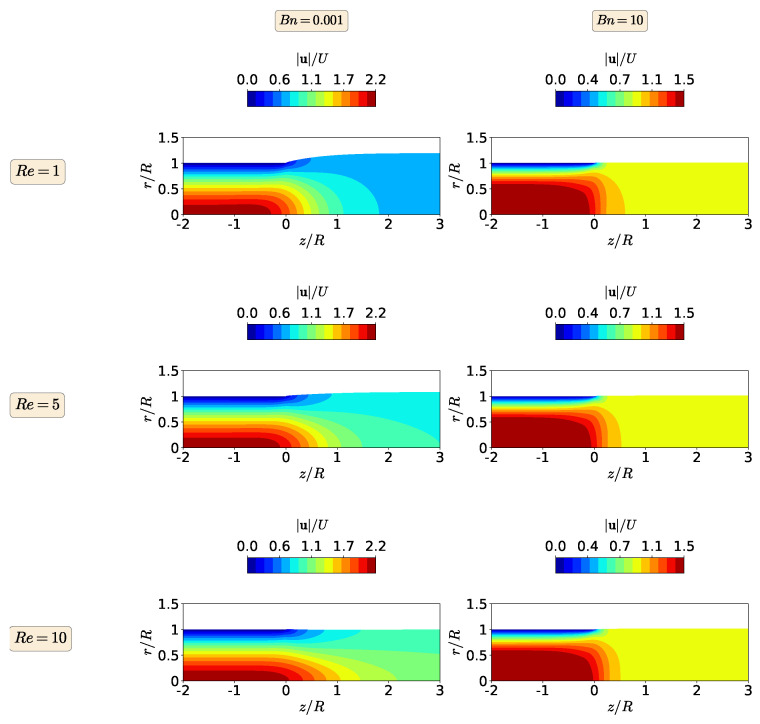
Contours of the magnitude of the polymer velocity vector at steady state for the isothermal extrudate swell of shear-thickening Herschel–Bulkley fluids (n=1.5) when Re=1 (**top**), Re=5 (**middle**) and Re=10 (**bottom**), and Bn=0.001 (**left**) and Bn=10 (**right**).

Figure 8 displays the contours of the magnitude of the polymer velocity vector for the isothermal extrudate swell of shear-thickening fluids (n=1.5), when Re=1,5, and 10 and Bn=0.001 and 10. As can be seen by the velocity contours at Bn=0.001, the maximum value for the magnitude of the polymer velocity vector is 2.2 times the inlet velocity for 1≤Re≤10, which is 10% larger than the value obtained for the Bingham flow (n=1), leading to variations of 17% in the extrudate swell ratio when Re is increased from 1 to 10. At the highest Bingham number (Bn=10), increasing the inertia effects does not change the extrudate swell ratio and the maximum value of the polymer velocity vector magnitude. The later remains 1.5 times the inlet velocity, which is approximately 13% larger than the one obtained for the Bingham fluid (n=1).

**Figure 9:**
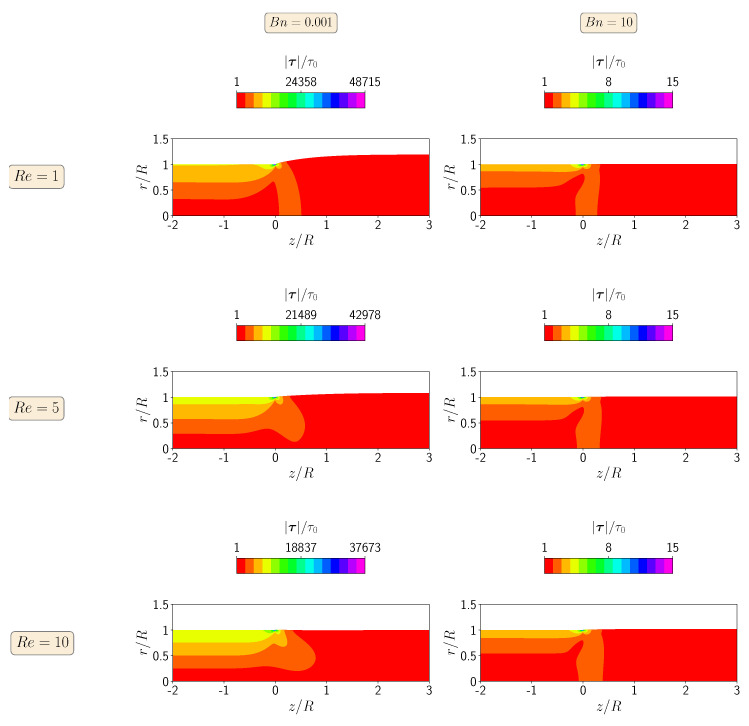
Contours of the magnitude of the polymer stress tensor at steady-state for the isothermal extrudate swell of shear-thickening Herschel–Bulkley fluids (n=1.5) when Re=1 (**top**), Re=5 (**middle**) and Re=10 (**bottom**), and Bn=0.001 (**left**) and Bn=10 (**right**).

Finally, in Figure 9, we show the steady-state contour plots of the magnitude of the polymer stress tensor for the isothermal extrudate swell of shear-thickening Herschel–Bulkley fluids (n=1.5) when Re=1,5, and 10 and Bn=0.001 and 10. As before, the stress contours follow the same trend as in the other fluids, i.e., the maximum magnitude of the polymer stress tensor decreases with an increase in inertia at Bn=0.001, while it is constant for the yield-stress-dominated flow (Bn=10), regardless of the enhancement of inertia from Re=1 to 10. Note also that the maximum magnitude of the polymer stress tensor for the shear-thickening Herschel–Bulkley fluid is around twice larger than the value obtained for the Bingham fluid at both Bn numbers (Bn=0.001 and 10).

### 4.4. Non-Isothermal and Yield Stress Effects in the Extrudate Swell of Bingham Fluids

In this section, we study the effects of temperature and yield stress in the extrudate swell ratio of Bingham fluids (n=1) at Reynolds number Re=10. We consider two different scenarios for the die wall temperature $T_{w}$, one where $T_{w}<T_{inlet}$ (cold wall) and another where $T_{w}>T_{inlet}$ (hot wall). We will then examine the thermally induced swelling behavior as the Bingham number increases for these two configurations.

The thermal and physical properties of a typical polystyrene [7] used in the simulations are listed in Table 1. An important issue for modeling the cooling of the polymer when it is extruded is the definition of the boundary condition at the polymer and air interface. In this work, we employed a defined convective heat flux as given in Equation (12). Additionally, the influence of the temperature on the rheological behavior of the material is controlled by the WLF equation for the shift factor employed in the temperature-dependent consistency parameter *k* (see Equations (6) and (7)). Typical extreme sets of WLF parameters ($c_{1},\;c_{2}$) are (4.54, 150.36), leading to thermorheological coupling [42].

In this case study, the ratio of moment diffusivity and thermal diffusivity of the fluid, defined by the dimensionless Prandtl number, is given by Pr=0.7 (see Equation (23)). A small Pr number means that heat diffuses very easily compared to moment.

**Figure 10:**
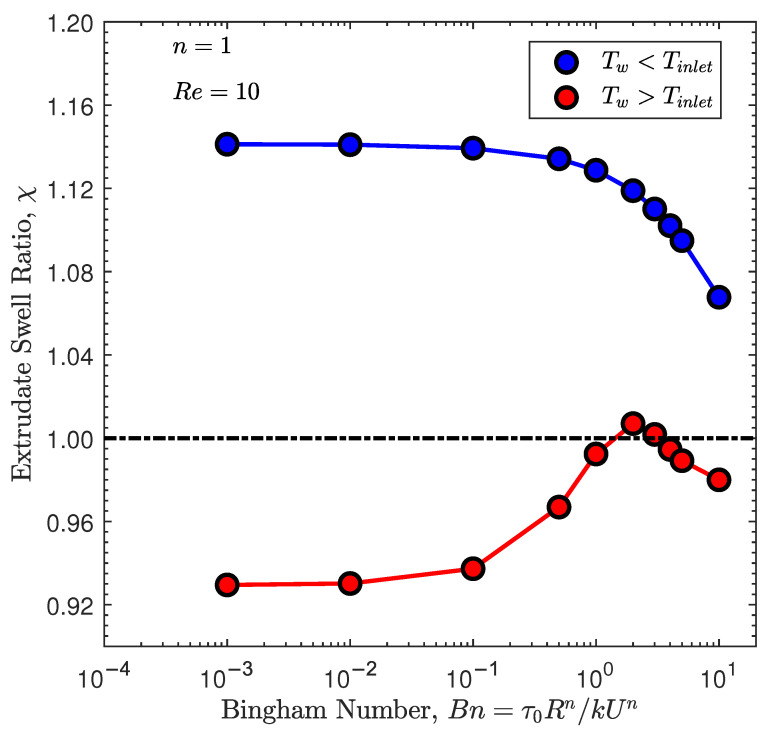
Steady-state extrudate swell ratio χ for the non-isothermal axisymmetric extrudate swell of Bingham fluids (n=1) at Re=10 and $Bn=\{10^{-3},10^{-2},10^{-1},0.5,1,2,3,4,5,10\}$.

In Figure 10, we show the extrudate swell ratio obtained by the newly developed solver for the non-isothermal flow of Bingham fluids at Re=10 and 0.001≤Bn≤10, with a cold (blue symbols) and hot (red symbols) die wall. For the cold wall, the extrudate swell ratio has a decreasing monotonic behavior, but being always larger than unity, meaning that the polymer expands after the die wall. Note that the decreasing monotonic behavior is related to the enhancement of the yield stress effects. Specifically, the extrudate swell ratio χ slightly decreases from Bn=0.001 to Bn=0.1, and then it falls with a sharper slope, having its smallest value around 1.06 at Bn=10. In contrast with the cold die wall, the extrudate swell ratio for the heating case study follows the same trend as the isothermal studies, where we first see an increase in the extrudate swell ratio followed up by a decrease due to the larger yield stress effects. Only at Bn=2 and 3, the polymer expands after the die wall, but for all the other Bn numbers, the polymer melt contracts, which is a similar behavior to the shear-thinning isothermal extrudate swell at Re=10 presented in Section 4.3. Note that in Labsi et al. [43], it is shown that neglecting the viscosity’s temperature dependency leads to undervaluing hydrodynamic properties, especially in the cooling case. If we compare the results shown in Figure 2 and Figure 10 at n=1 and Re=10, we see that the extrudate swell ratio for the case neglecting viscosity’s temperature dependency (Figure 2) is smaller than the one obtained when viscosity’s temperature dependence is taken into account (Figure 10), and this behavior is more noticeable for the cooling case, corroborating the conclusion presented in Labsi et al. [43].

**Figure 11:**
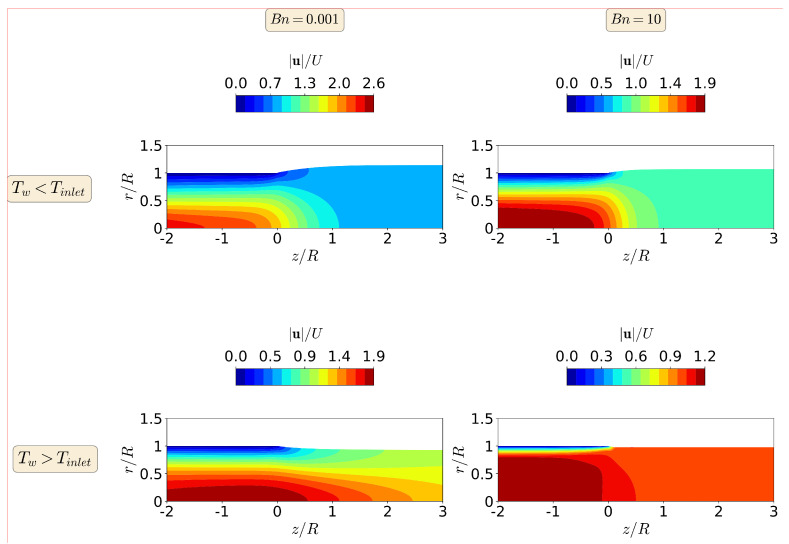
Contours of the magnitude of the polymer velocity vector at steady-state for the non-isothermal asymmetric extrudate swell flow of Bingham fluids (n=1) at Re=10 for Bn=0.001 (**left**) and Bn=10 (**right**), when $T_{w}<T_{inlet}$ (**top**) and $T_{w}>T_{inlet}$ (**bottom**).

Figure 11 shows the magnitude of the polymer velocity vector at Re=10 for Bn=0.001 and 10, and for the cold and hot die wall case studies. For Bn=0.001 (negligible yield-stress effects), the maximum value of the magnitude of the polymer velocity vector is 30% higher in the cold die wall case study and 5% lower in the hot die wall case study, than the maximum values obtained for isothermal conditions. When the higher Bn number is considered (Bn=10), the maximum value of the magnitude of the polymer velocity vector is 46% higher in the cold die wall case study and 8% lower in the hot die wall case study, than the maximum values obtained for isothermal conditions. Notice also that for the cold die wall case study at Bn=10 the velocity profile has changed from a plug-flow shape in isothermal conditions to a parabolic profile shape now. This means that the non-isothermal effects are stronger than the yield stress effects for the cold die walls case study at Re=10. For the hot die wall case study at Bn=10, the velocity profile maintains the plug-flow shape profile, leading to a reduction in the extrudate die-swell ratio.

**Figure 12:**
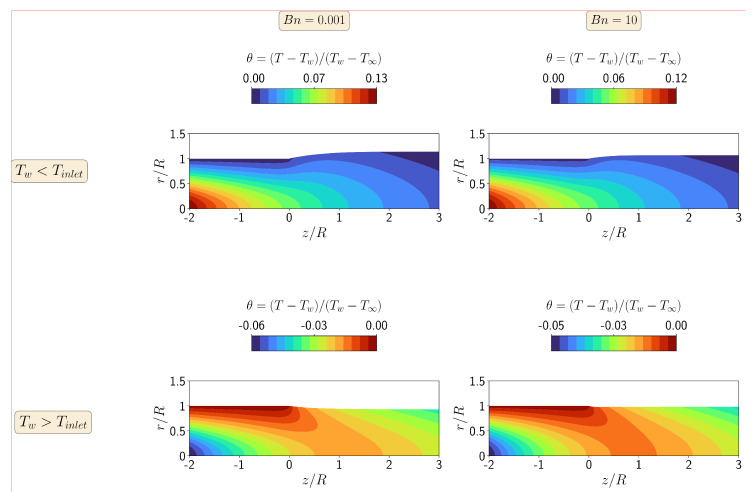
Contours of dimensionless temperature at steady-state for the non-isothermal asymmetric extrudate swell flow of Bingham fluids (n=1) at Re=10 for Bn=0.001 (**left**) and Bn=10 (**right**), when $T_{w}<T_{inlet}$ (**top**) and $T_{w}>T_{inlet}$ (**bottom**).

In Figure 12, we show contours of the steady-state dimensionless temperature field at Re=10 for Bn=0.001 and 10, and for the cold and hot die wall case studies. As can be seen in Figure 12, the temperature variation Δθ for the case where cold die walls are used in the simulations is approximately twice the one obtained when hot die walls are employed, which will not induce a high swelling ratio for the latter case (hot die walls). This is due to the fact that the material flows toward the cooler (more viscous) region as the flow rearranges in the extrudate, which will give higher swelling ratio of the cooler side (as shown in the cold die walls).

## 5. Conclusions

A numerical algorithm able to solve non-isothermal and inelastic non-Newtonian free-surface flows based on the Arbitrary Lagrangian–Eulerian (ALE) formulation was presented and implemented using the finite-volume method. The implementation was performed in the open-source OpenFOAM framework [26], where the interface is tracked in a semi-implicit manner, which allowed robust and stable deformations of the interface.

The newly developed algorithm was assessed in terms of accuracy for isothermal flow simulations of the axisymmetric extrudate swell of Bingham and Herschel–Bulkley fluids. The effects of inertia and yield stress on the extrudate swell ratio computed by the newly developed algorithm were studied and compared with results found in the scientific literature for the range of the dimensionless Reynolds (Re) and Bingham (Bn) numbers as follows: 1≤Re≤10 and 0.001≤Bn≤10. Additionally, the contours for the magnitude of the polymer velocity vector and stress tensor fields are also shown and discussed. For the isothermal Bingham flows, the extrudate swell ratio was found to vary by approximately 13% from the lowest to the highest Re number when the yield stress effects are negligible (Bn=0.001). Additionally, at higher Bn numbers, the yield stress effects are dominant and the extrudate swell ratio is equal for all the Re numbers tested, being lesser than unity, which means that the swell contracts. For the isothermal Herschel–Bulkley flows of the shear-thinning fluid, the extrudate swell ratio was found to vary by approximately 8% from the lowest to the highest Re number when the yield stress effects are negligible (Bn=0.001). On the other hand, at higher Bn numbers, the yield stress effects are dominant and the extrudate swell ratio is equal for all the Re numbers tested, being also lesser than unit as in the Bingham flow case, meaning that the swell contracts. In the case of using a shear-thickening fluid, the extrudate swell ratio was found to vary by approximately 17% from the lowest to the highest Re number when the yield stress effects are negligible (Bn=0.001). Additionally, at higher Bn numbers, the extrudate swell ratio approaches the unitary value, which means that the polymer melt neither contracts nor expands.

Finally, the newly developed algorithm was applied to study the non-isothermal flow of axisymmetric extrudate swell using Bingham fluids at Re=1. The effects of temperature and yield stress on the extrudate swell ratio computed by the newly developed algorithm were analyzed for the range of the dimensionless Bingham (Bn) numbers, 0.001≤Bn≤10, and two different configurations, one representing a cold die wall and the other an hot die wall. Additionally, the contours for the magnitude of the polymer velocity vector and temperature fields are shown and discussed. For the cold die wall it was found that the extrudate swell ratio has a monotonic decreasing behavior but always with a value greater than unity for 1≤Bn≤10. However, for the hot die wall, the extrudate swell ratio first increases for 0.001≤Bn≤2 and then decreases for 2≤Bn≤10, being greater than unity only at Bn=2 and 3.

In summary, the results presented here show that the newly developed interface tracking code can accurately predict the non-isothermal extrudate swell of inelastic non-Newtonian matrix-based fluids. The code that was implemented here is being currently extended to handle viscoelastic non-Newtonian fluid flow calculations.

## Figures and Tables

**Table 1:** General conditions used in the non-isothermal simulations of the extrudate swell of Bingham fluids (n=1).

$k_{p}$ (W/m °C)	0.18
ρ (kg/m${}^{3}$)	1400
$c_{p}$ (J/kg °C)	1000
Inlet profile temperature, $T_{inlet}$ (°C)	180
Die wall temperature, $T_{w}$ (°C)	140 or 220
Room temperature, $T_{\infty }$ (°C)	20
Air convection heat transfer	
coefficient (free convection), *h* (W/m${}^{2}$ °C)	5

## References

[1] Mirjalili S., Jain S.S., Dodd M. (2017). Interface-Capturing Methods for Two-Phase Flows: An Overview and Recent Developments.

[2] Nie Y., Hao J., Lin Y.-J., Sun W. (2018). 3D simulation and parametric analysis of polymer melt flowing through spiral mandrel die for pipe extrusion. Adv. Polym. Technol..

[3] Fernandes C., Pontes A.J., Viana J.C., Nóbrega J.M., Gaspar-Cunha A. (2014). Modeling of Plasticating Injection Molding—Experimental Assessment. Int. Polym. Process..

[4] Pedro J., Ramôa B., Nóbrega J.M., Fernandes C. (2020). Verification and Validation of openInjMoldSim, an Open-Source Solver to Model the Filling Stage of Thermoplastic Injection Molding. Fluids.

[5] Tadmor Z., Klein I. (1968). Computer Programs for Plastic Engineers.

[6] Agur E.E., Vlachopoulos J. (1982). Numerical Simulation of a Single-Screw Plasticating Extruder. Polym. Eng. Sci..

[7] Habla F., Fernandes C., Maier M., Densky L., Ferrás L.L., Rajkumar A., Carneiro O.S., Hinrichsen O., Nóbrega J.M. (2016). Development and validation of a model for the temperature distribution in the extrusion calibration stage. Appl. Therm. Eng..

[8] Rajkumar A., Ferrás L.L., Fernandes C., Carneiro O.S., Becker M., Nóbrega J.M. (2017). Design guidelines to balance the flow distribution in complex profile extrusion dies. Int. Polym. Process..

[9] Vlachopoulos J., Polychronopoulos N.D. (2019). Understanding Rheology and Technology of Polymer Extrusion.

[10] Vlcek J., Vlachopoulos J. (1989). Effect of Die Wall Cooling or Heating on Extrudate Swell. Polym. Eng. Sci..

[11] Phuoc H.B., Tanner R.I. (1980). Thermally-induced extrudate swell. J. Fluid Mech..

[12] Karagiannis A., Hrymak A.N., Vlachopoulos J. (1989). Three-dimensional non-isothermal extrusion flows. Rheol. Acta.

[13] Spanjaards M.M.A., Hulsen M.A., Anderson P.D. (2020). Computational analysis of the extrudate shape of three-dimensional viscoelastic, non-isothermal extrusion flows. J. Non–Newton. Fluid Mech..

[14] Georgiou G.C., Boudouvis A.G. (1999). Converged solutions of the Newtonian extrudate-swell problem. Int. J. Numer. Methods Fluids.

[15] Fakhari A., Tukovic Z., Carneiro O.S., Fernandes C. (2021). An Effective Interface Tracking Method for Simulating the Extrudate Swell Phenomenon. Polymers.

[16] Bingham E.C. (1922). Fluidity and Plasticity.

[17] Herschel W.H., Bulkley R. (1926). Konsistenzmessungen von Gummi-Benzollösungen. Kolloid D.

[18] Papanastasiou T.C. (1987). Flows of materials with yield. J. Rheol..

[19] Ellwood K.R.J., Georgiou G.C., Papanastasiou T.C., Wilkes J.O. (1990). Laminar jets of Bingham plastic liquids. J. Rheol..

[20] Frigaard I.A., Nouar C. (2005). On the usage of viscosity regularization methods for visco-plastic fluid flow computation. J. Non–Newton. Fluid Mech..

[21] Beverly C.R., Tanner R.I. (1989). Numerical analysis of extrudate swell in viscoelastic materials with yield stress. J. Rheol..

[22] Hurez P., Tanguy P.A., Bertrand F.H. (1990). A finite element analysis of dieswell with pseudoplastic and viscoplastic fluids. Comput. Methods Appl. Mech. Eng..

[23] Abdali S.S., Mitsoulis E., Markatos N.C. (1992). Entry and exit flows of Bingham fluids. J. Rheol..

[24] Mitsoulis E., Abdali S.S., Markatos N.C. (1993). Flow simulation of Herschel–Bulkley fluids through extrusion dies. J. Chem. Eng..

[25] Kountouriotis Z., Georgiou G.C., Mitsoulis E. (2014). Numerical study of the combined effects of inertia, slip, and compressibility in extrusion of yield stress fluids. Rheol. Acta.

[26] OpenCFD (2007). OpenFOAM—The Open Source CFD Toolbox—User’s Guide.

[27] Tuković Ž., Jasak H. (2012). A moving mesh finite volume interface tracking method for surface tension dominated interfacial fluid flow. Comput. Fluids.

[28] Zhou Y.-G., Wu W.-B., Zou J., Turng L.-S. (2016). Dual-scale modeling and simulation of film casting of isotactic polypropylene. J. Plast. Film Sheeting.

[29] Tuković Z., Jasak H. Simulation of free-rising bubble with soluble surfactant using moving mesh finite volume/area method. Proceedings of the 6th International Conference on CFD in Oil & Gas, Metallurgical and Process Industries.

[30] Demirdžić I., Perić M. (1988). Space conservation law in finite volume calculations of fluid flow. Int. J. Numer. Methods Fluids.

[31] Ferziger J.H., Peric M. (2002). Computational Methods for Fluid Dynamics.

[32] Van Doormaal J.P., Raithby G.D. (1984). Enhancement of the SIMPLE method for predicting incompressible fluid flows. Numer. Heat Transf..

[33] Issa R.I. (1986). Solution of the implicitly discretised fluid flow equations by operator-splitting. J. Comput. Phys..

[34] Tuković Ž., Perić M., Jasak H. (2018). Consistent second-order time-accurate non-iterative PISO-algorithm. Comput. Fluids.

[35] Moukalled F., Mangani L., Darwish M. (2016). The Finite Volume Method in Computational Fluid Dynamics: An Advanced Introduction with OpenFOAM and Matlab.

[36] Tuković Ž., Karač A., Cardiff P., Jasak H. (2018). OpenFOAM Finite Volume Solver for Fluid-Solid Interaction. Trans. Famena.

[37] Rhie C.M., Chow W.L. (1983). Numerical study of turbulent flow past an isolated airfol with trailing edge separation. AIAA J..

[38] Jacobs D. (1980). Preconditioned Conjugate Gradient Methods for Solving Systems of Algebraic Equations.

[39] Ajiz M., Jennings A. (1984). A robust incomplete Cholesky-conjugate gradient algorithm. J. Numer. Meth. Eng..

[40] Lee J., Zhang J., Lu C.-C. (2003). Incomplete LU preconditioning for large scale dense complex linear systems from electromagnetic wave scattering problems. J. Non–Newton. Fluid Mech..

[41] Kountouriotis Z., Georgiou G.C., Mitsoulis E. (2013). On the combined effects of slip, compressibility, and inertia on the Newtonian extrudate-swell flow problem. Comput. Fluids.

[42] Peters G.W.M., Baaijens F.P.T. (1997). Modelling of non-isothermal viscoelastic flows. J. Non–Newton. Fluid Mech..

[43] Labsi N., Benkahla Y.K., Boutra A. (2017). Temperature-dependent shear-thinning Herschel–Bulkley fluid flow by taking into account viscous dissipation. J. Braz. Soc. Mech. Sci. Eng..

